# The effects and interrelationships of intercropping on Cotton Verticillium wilt and soil microbial communities

**DOI:** 10.1186/s12866-023-02780-6

**Published:** 2023-02-13

**Authors:** Yun Zhang, Yuanxue Yang, Xiuyun Lu, Aiyu Wang, Chao Xue, Ming Zhao, Jianhua Zhang

**Affiliations:** 1 Institute of Industrial Crops, Shandong Academy of Agricultural Sciences, Jinan, 250100 China; 2 Institute of Plant Protection, Hebei Academy of Agricultural and Forestry Sciences, Baoding, 071000 China

**Keywords:** Interplanting, Cotton Verticillium wilt, Soil, Microbial community structure

## Abstract

**Background:**

Cotton Verticillium wilt, causing by *Verticillium dahliae*, has seriously affected the yield and quality of cotton. The incidence of Verticillium wilt in cotton fields has been on the rise for many years, especially after straw has been returned to the fields. Intercropping can reduce the incidence of soil borne diseases and is often used to control crop diseases, but the relationship between the effects of intercropping on microbial communities and the occurrence of plant diseases is unclear. This research explored the relationship between soil microbial community structure and Cotton Verticillium wilt in interplanting of cotton-onion, cotton-garlic, cotton-wheat and cotton monocultures. Amplicon sequencing applied to the profile of bacterial and fungal communities.

**Results:**

The results showed that the disease index of Cotton Verticillium wilt was significantly reduced after intercropping with cotton-garlic and cotton-onion. Chao1 and Sobs indices were not significantly different in the rhizosphere soil and pre-plant soils of the four planting patterns, but the pre-plant fungal shannon index was significantly lower in the cotton-onion intercropping plot than in the other three plots. PCoA analysis showed that the soil microbial communities changed to a certain extent after intercropping, with large differences in the microbial communities under different cropping patterns. The abundance of *Chaetomium* was highest in the cotton-garlic intercropping before planting; the abundance of *Penicillium* was significantly higher in the cotton-wheat intercropping than in the other three systems.

**Conclusion:**

Cotton-garlic and cotton-onion interplanting can control Cotton Verticillium wilt by affecting the soil microbial community. Fungi of the genera *Chaetomium* and *Penicillium* may be associated with plant disease resistance.

## Background

Verticillium wilt is one of the most serious cotton diseases in the world [1], and is mainly caused by *Verticillium dahliae* Kleb. At present, the main method to control the disease is to select resistant varieties. However, due to a lack of effective sources of resistance, no highly resistant varieties have yet been successfully bred. Soil chemical fumigation is effective, but costly, polluting and difficult to operate in the field. Biological control is currently one of the most promising methods to control Verticillium wilt, with the advantages of being safe to use and environmentally friendly [2, 3]. Many microorganisms have been proved to possess biological control effects and have been applied in the field [4]. Changes in biotic and abiotic factors in the field have an impact on the survival and colonization of biocontrol bacteria or fungi. Therefore, successful biological control products against soil-borne diseases in the field production of commercial crops are still very limited [5].

The raising of plant diversity in the ecosystem can enhance its stability and avoid its vulnerability to biotic and abiotic stresses [6]. Crop monoculture is very detrimental to control the occurrence of diseases. Cotton monoculture favours the development of ramularia leaf spot [7]; long-term vanilla monoculture often leads to the occurrence of Fusarium wilt [8]; replanting (continuous monoculture) disease seriously affected productivity and quality of *Rehmannia glutinosa* [9]. Increasing the diversity of farmland species can effectively reduce crop disease hazards, significantly reduce the use of chemical fungicides, reduce environmental pollution, improve the quality of agricultural products and achieve sustainable agricultural development [10]. In recent years, there has been an increasing amount of research into the use of intercropping for disease control. The incidence and severity of tomato Verticillium wilt could be reduced by growing tomatoes and potatoes together with onions [11]; intercropping maize and pepper suppressed pepper blight [12]; intercropping rice and watermelon suppressed watermelon Fusarium wilt [13]; intercropping maize and soybean reduced soybean red crown rot damage [14]. Intercropping has received a lot of attention for its good effect on controlling soil-borne diseases.

Intercropping suppresses soil diseases in two ways, either by improving crop resistance or by reducing the invasion of pathogenic bacteria [15, 16]. It has been shown that intercropping reduces soil-borne diseases by increasing plant resistance to pathogens, while intercropping wheat and watermelon reduced the incidence of watermelon wilt, induced an increase in phenylalanine ammonia lyase (PAL) and polyphenol oxidase (PPO) activities, and increased the content of flavonoids, total soluble phenols and lignin [17]. Plant–microbe interactions help to promote plant growth, resist disease in a changing environment and achieve sustainable agriculture without disrupting ecosystem function. Rhizosphere soil microbial diversity maintains soil health and productivity [18]. Mustard-cucumber intercropping significantly increased the diversity of soil bacterial and fungal communities and the abundance of beneficial microorganisms [19]; peanut-tobacco intercropping improved soil ecology and microbial community structure, increased the number of beneficial bacteria and reduced the number of pathogens [20]. In conclusion, soil microbial communities play a very important role in the resistance of intercropping patterns to soil-borne diseases.

In previous studies, our research found that intercropping cotton with wheat, onion and garlic reduced the incidence of Cotton Verticillium wilt [21], but the effects of these intercropping patterns on soil microbial communities and the abundance of beneficial microorganisms are not known. The aim of this study was to elucidate the effects of three intercropping patterns of cotton-wheat, cotton-onion and cotton-garlic on soil microbial communities and the relationship between soil microbial community structure and Cotton Verticillium wilt.

## Materials and methods

### Crop varieties

The cotton variety was Verticillium wilt resistant variety Lumian 1141, the wheat variety was Jimai 22, the onion variety was Dongfanghong 6, and the garlic variety was Jinxiang garlic.

### Interplanting experiment

This experiment was conducted in Yangzhuang Village, Juye County, Heze City, Shandong Province (116°48′E, 35°13′N). The soil type in this area was fluvial soil, which was neutral or slightly alkaline. The climate was temperate continental, with long dry and cold winters, hot summers and frequent floods, and late autumn droughts. The experiment was conducted in four plots, namely, cotton-wheat interplanting, cotton-onion interplanting, cotton-garlic interplanting and cotton monoculture. These plots repeated the same planting pattern for more than three years.

Cotton-wheat interplanting (CW): wheat was first planted in the autumn. According to the 4–2 mode, the width was 150 cm, four rows of wheat were planted the following year with a row spacing of 20 cm. Two rows of cotton were planted with a row spacing of 40 cm, covered with plastic film. The narrow rows of cotton were 40 cm, the wide rows were 110 cm, and the spacing between wheat and cotton was 25 cm.

Cotton-garlic intercropping (CG): adopted 4–2 mode with a bandwidth of 150 cm. In autumn, four rows of garlic were planted with a row spacing of 20 cm. Two rows of cotton was planted with a row spacing of 40 cm the following year, and the spacing between garlic and cotton was 25 cm.

Cotton-onion interplanting (CO): in the autumn, the 4–2 mode was adopted, with a bandwidth of 150 cm, four rows of onion were planted, with a row spacing of 20 cm. Two rows of cotton were planted the following year, with a narrow row of 40 cm, a wide row of 110 cm, and a spacing of 25 cm between onion and cotton.

**Fig. 1:**
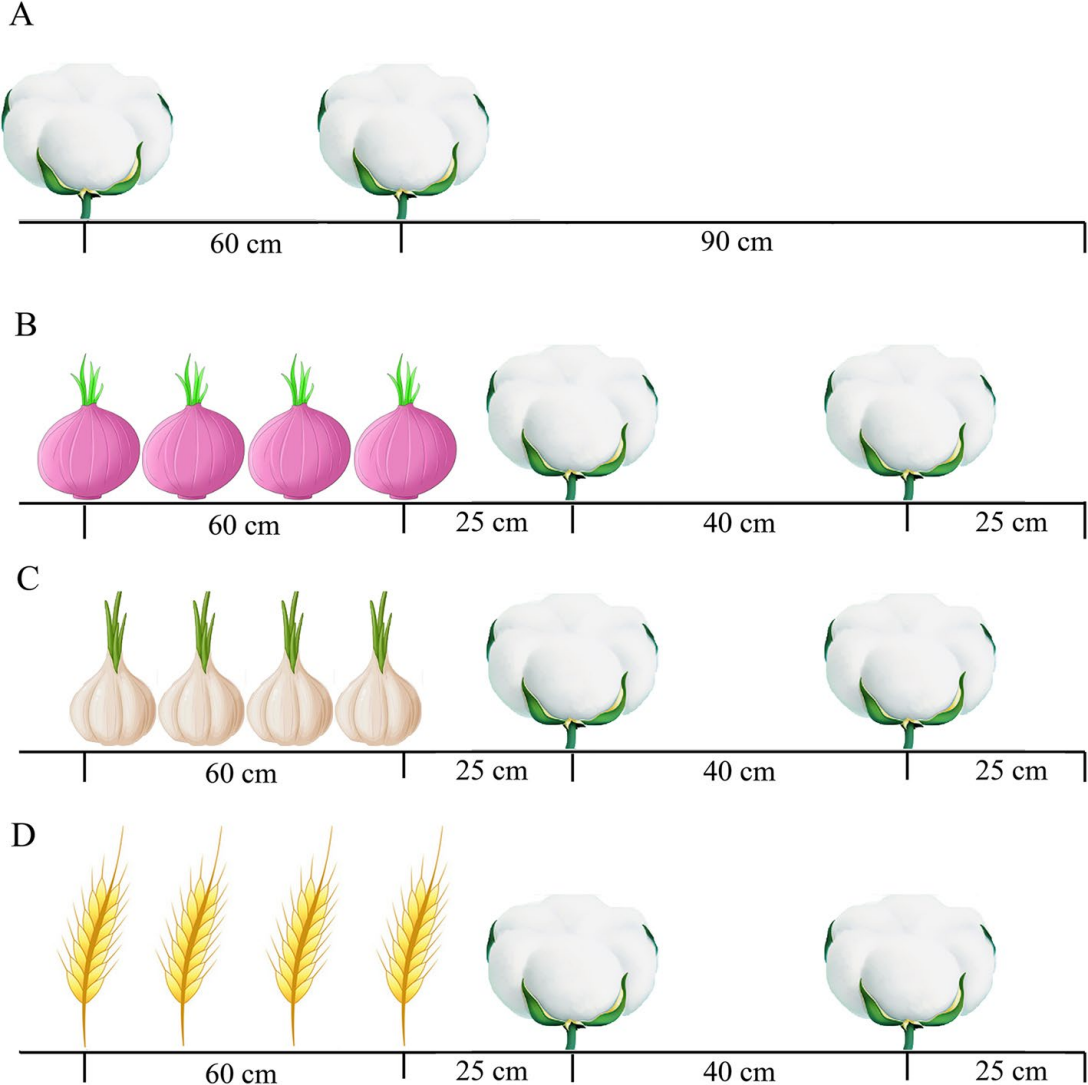
Sketch of field experiments in cropping patterns. **A** Cotton monoculture pattern, **B** Cotton-onion intercropping pattern, **C** Cotton-garlic intercropping pattern, **D** Cotton-wheat intercropping pattern

Cotton monoculture (C): after harvesting cotton in the autumn, no crops were planted. Cotton was planted according to the spacing between rows the following year, with a narrow row of 60 cm and a wide row of 90 cm (Fig. 1).

### Incidence degree of Cotton Verticillium wilt in different planting patterns

Cotton Verticillium wilt was investigated by the diagonal 5-point survey method in the four fields, C, CO, CW, CG, respectively. The disease index was investigated according to the 5-level classification standard referred to Wei et al. (2019) method [22], and the disease index (DI) = (0·n_0_ + 1·n_1_ + 2·n_2_ + 3·n_3_ + 4·n_4_) / (4·n) × 100%. n_0_—n_4_ was the number of plants with corresponding disease grade (0–4), and n was the total number of plants investigated.

### Soil sampling

In the autumn, before planting wheat, onion and garlic, soil samples were taken from cotton monoculture, cotton-wheat intercropping, cotton-onion intercropping and cotton-garlic intercropping plots, respectively. The sampling method refered to the published process [23]. A total of 12 samples were taken as a control, which were Cotton-1 (C-1), C-2, C-3, Cotton-Wheat-1 (CW-1), CW-2, CW-3, Cotton-Onion-1 (CO-1), CO-2, CO-3 Cotton-Garlic-1 (CG-1), CG-2 and CG-3. After planting cotton the following year, the rhizosphere soil of cotton plants infected by *V. dahliae* was taken according to previous sampling method during the onset period of Cotton Verticillium wilt [23]. Take 12 samples, including Cotton-rhizosphere 1 (C-r-1), C-r-2, C-r-3, Cotton-wheat-rhizosphere-1 (CW-r-1), CW-r-2, CW-r-3, Cotton-Onion-rhizosphere-1 (CO-r-1), CO-r-2, CO-r-3, Cotton-Garlic-rhizosphere-1 (CG-r-1), CG-r-2 and CG-r-3.

### Soil sequencing analysis

DNA was extracted from 24 soil samples (above) according to the FastDNA™ SPIN Kit for Soil DNA Extraction Kit specification. After the DNA quality and DNA concentration was detected, the 24 DNA were sequenced by fungal ITS and bacterial 16S. The bacterial 16S rDNA primer and the fungal ITS sequencing primer was referred to Fadrosh et al. (2014) and Karlsson et al. (2014) [24, 25]. PCR reaction system and program were referred to the method of Xiong et al. (2015) [26]. The processing of PCR products, sequencing, operational taxonomic units (OTUs) clustering, fungal and bacterial species annotation were referred to previously published methods [23].

### Statistical analysis

Alpha diversity were calculated by analysing the Observed species (Sobs), shannon, and Chao 1 indices by the QIIME 2 process [27], the pictures were drawn by R (v3.5.2) package, and the R language ade4 package (1.7.13) was used for principal coordinate analysis (PCoA) analysis. One-way analysis of variance (ANOVA) was used to determine the difference of disease index, and Tukey's HSD method was used to test.

## Results

### Disease index of Cotton Verticillium wilt under different planting patterns

**Fig. 2:**
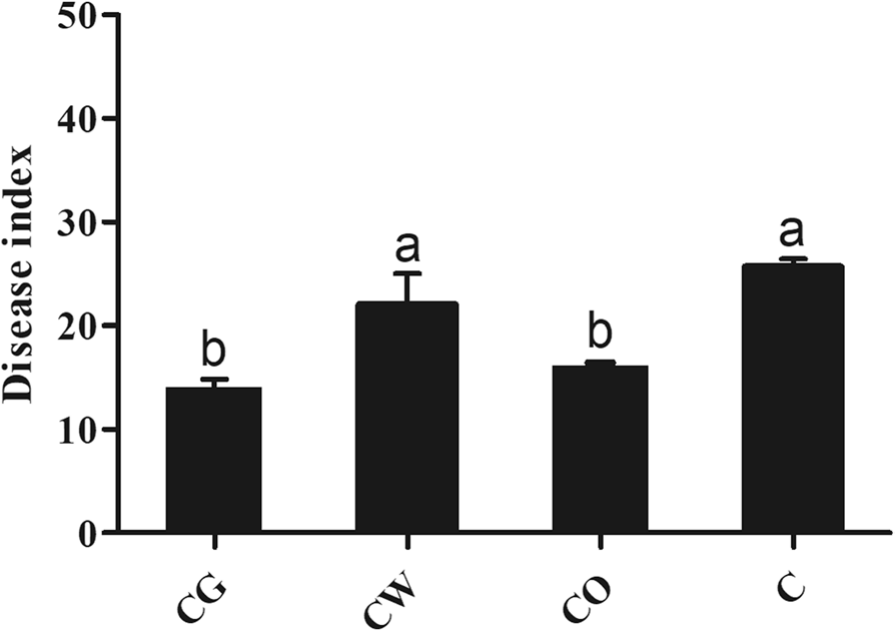
Disease index of Cotton Verticillium wilt. Data are mean ± SE. Different letters indicate significant difference at the level of *p* < 0.05. C represented cotton monoculture, CW represented cotton-wheat intercropping, CG represented cotton-garlic intercropping, CO represented cotton-onion intercropping

The investigation results showed that the disease index of Cotton Verticillium wilt in the cotton-garlic intercropping and cotton-onion intercropping were 14.00 and 16.17, respectively, which was significantly lower than that in the cotton monoculture of 25.67 (Fig. 2); disease index in cotton-wheat intercropping also decreased, but compared with cotton monoculture, the difference was not significant. The results showed that cotton-garlic and cotton-onion intercropping could significantly reduce the incidence of Verticillium wilt.

### Alpha diversity

**Fig. 3:**
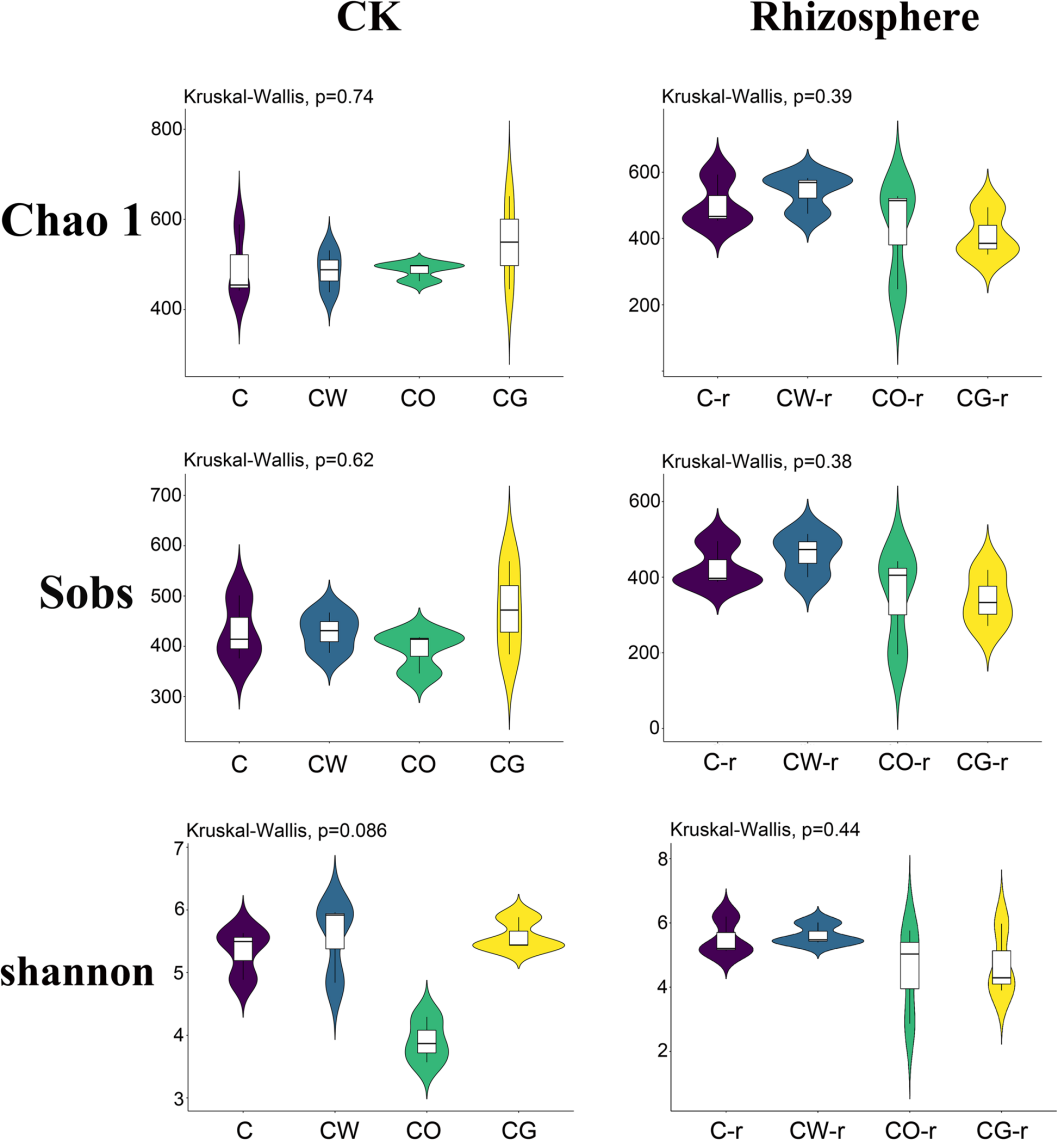
Fungal alpha diversity indices for the four different planting modes. C represented cotton monoculture, CW represented cotton-wheat intercropping, CG represented cotton-garlic intercropping, CO represented cotton-onion intercropping

In the pre-planting soil and cotton rhizosphere soil, the Chao 1, shannon and Sobs indices of fungi in the cotton-onion intercropping pattern were lower than those in the other three patterns, and the shannon index in the pre-planting soil was significantly lower than that in the other three plots. Chao 1, shannon and Sobs indices were the highest in the pre-planting cotton-garlic intercropping plots, which were higher than the cotton monoculture plot, but the difference was not significant. In the cotton rhizosphere soil, Chao 1, shannon and Sobs indices of cotton-wheat interplanting plots were the highest, which were higher than the cotton monoculture plot, but the difference was not significant (Fig. 3).

**Fig. 4:**
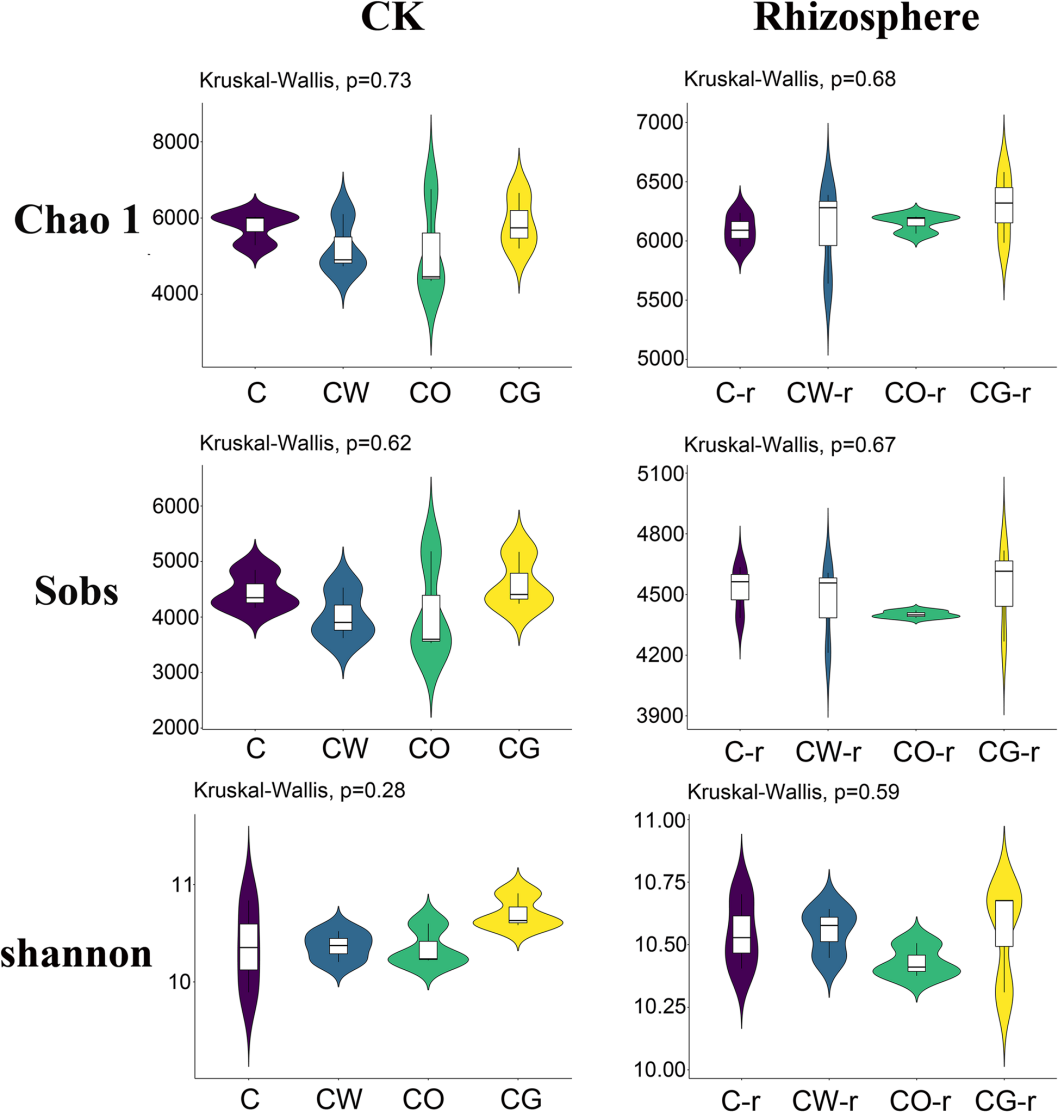
Bacterial alpha diversity indices for the four different planting modes. C represented cotton monoculture, CW represented cotton-wheat intercropping, CG represented cotton-garlic intercropping, CO represented cotton-onion intercropping

In the pre-planting soil and cotton rhizosphere soil, there was no difference in the bacterial Chao 1, shannon and Sobs indices of these four planting patterns. The three indices were the highest in the pre-planting soil of cotton-garlic plot, which were higher than the cotton monoculture plot. The Chao 1 index in cotton rhizosphere soil was the highest in cotton-onion interplanting, the shannon index was the highest in cotton-wheat interplanting, and the Sobs index was the highest in cotton monoculture plot (Fig. 4).

### Comparison of OTUs in the four different planting patterns

**Fig. 5:**
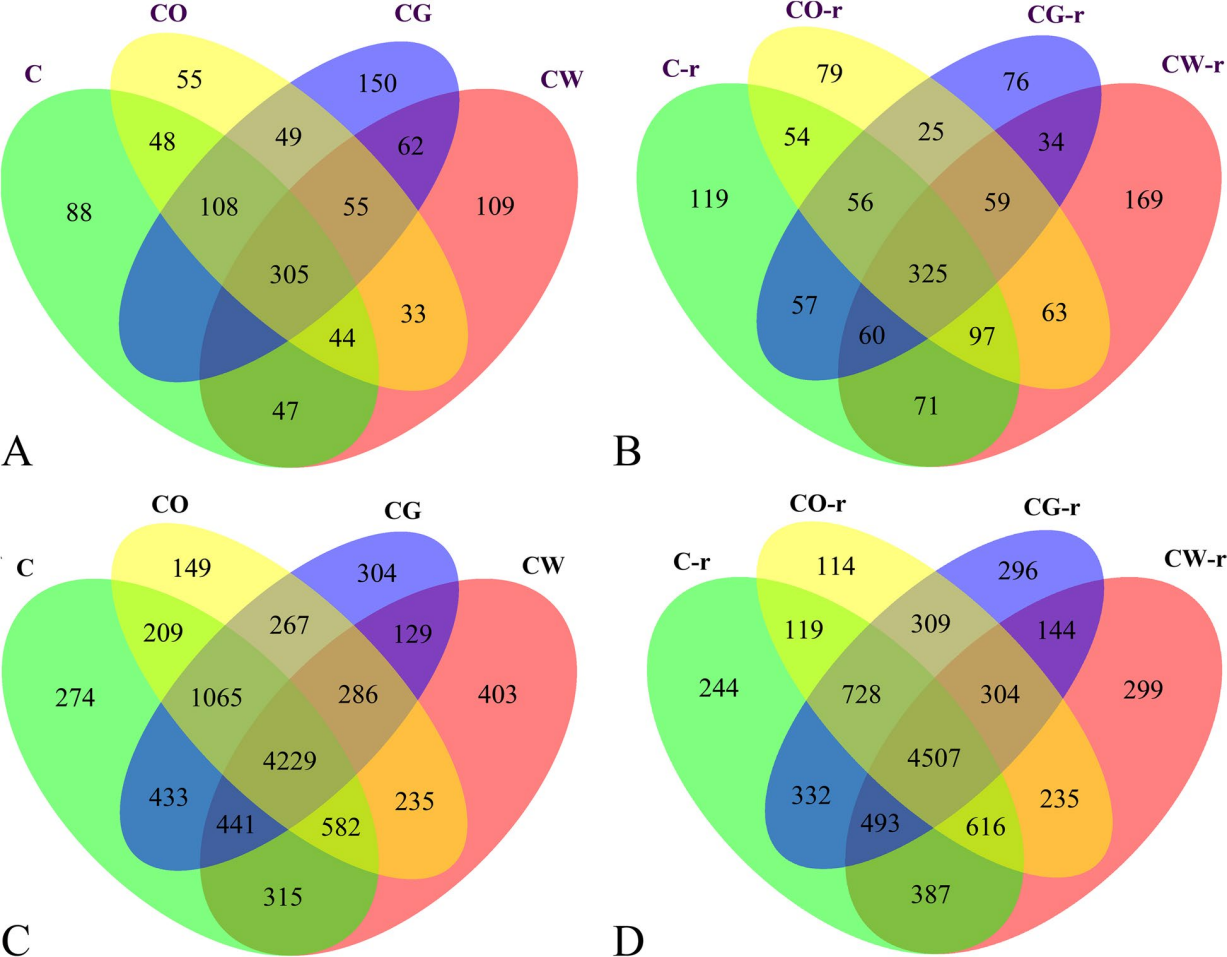
OTUs of soil fungi and bacteria in the four different planting patterns. A and C represented the OTU of fungi and bacteria in the soil before planting, respectively. B and D represented the OTU of fungi and bacteria in the rhizosphere soil of plants infected by *V. dahliae*, respectively. C represented cotton monoculture, CW represented cotton-wheat intercropping, CG represented cotton-garlic intercropping, CO represented cotton-onion intercropping

For fungi, before planting, there were 782 fungi OTUs in the pre-planting cotton monoculture plot, 871 in the cotton-garlic intercropping plot, 697 in the cotton-onion intercropping plot, and 726 in the cotton-wheat intercropping plot. There were 555 identical OTUs in the cotton monoculture and the cotton-garlic intercropping plots, 505 in the cotton-onion intercropping plot and 467 in the cotton-wheat intercropping plot. There were 55 unique and common OTUs in the interplanting plots. In the cotton rhizosphere soil, there were 532 common OTUs in the cotton monoculture plot and the cotton-onion intercropping plot, 498 in the cotton-garlic intercropping plot and 553 in the cotton-wheat intercropping plot. There were 59 unique and common OTUs in the interplanting plots (Fig. 5).

For bacteria, before planting, there were 7548 OTUs in cotton monoculture plot, 7154 in cotton-garlic intercropping plot, 7022 in cotton-onion intercropping plot and 6620 in cotton-wheat intercropping plot. There were 6168 common OTUs in cotton monoculture and cotton-garlic intercropping, 6085 in cotton-onion intercropping and 5567 in cotton-wheat intercropping. There were 286 unique and common OTUs in the interplanting plots. In the cotton rhizosphere soil, there were 5970 common OTUs in the cotton monoculture and the cotton-onion intercropping, 6060 in the cotton-garlic intercropping, and 6003 in the cotton-wheat intercropping. There were 304 unique and common OTUs in the interplanting plots (Fig. 5).

### PCoA analysis

**Fig. 6:**
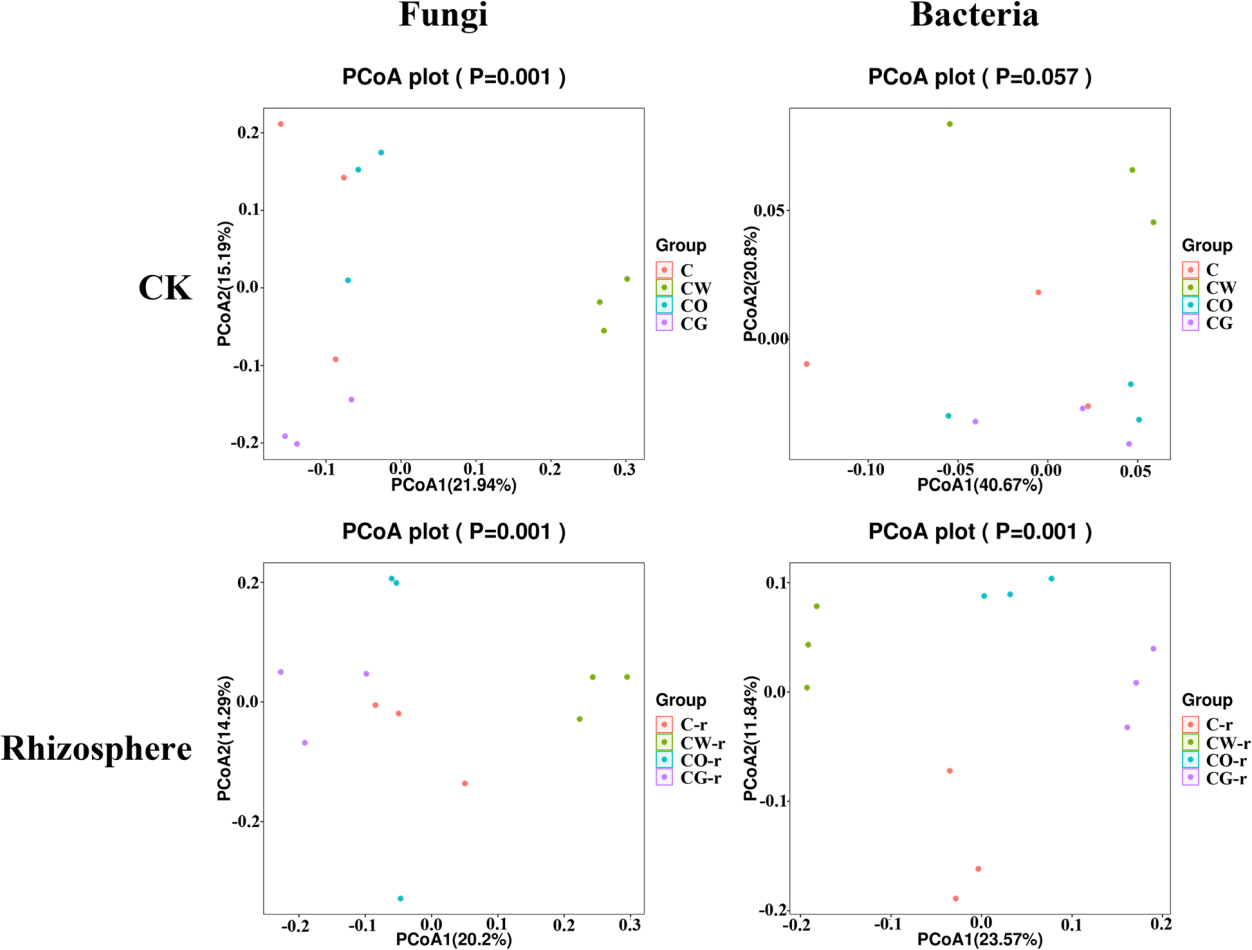
PCoA analysis of fungi and bacteria in the four different planting patterns. C represented cotton monoculture, CW represented cotton-wheat intercropping, CG represented cotton-garlic intercropping, CO represented cotton-onion intercropping

PCoA analysis based on OTU level showed that bacteria and fungi community in different planting patterns was different, and the three soil samples of the same planting pattern were not closely distributed (Fig. 6). In addition, before planting, there was no significant difference in fungal community between cotton monoculture and cotton-onion interplanting system, and there was no significant difference in bacterial community between cotton-onion interplanting and cotton-garlic interplanting. In the rhizosphere soil of cotton, the distribution of rhizosphere bacterial and fungal communities in different planting patterns was different. The distribution of rhizosphere bacteria in different interplanting patterns had obvious limits (Fig. 6).

### Community composition

**Fig. 7:**
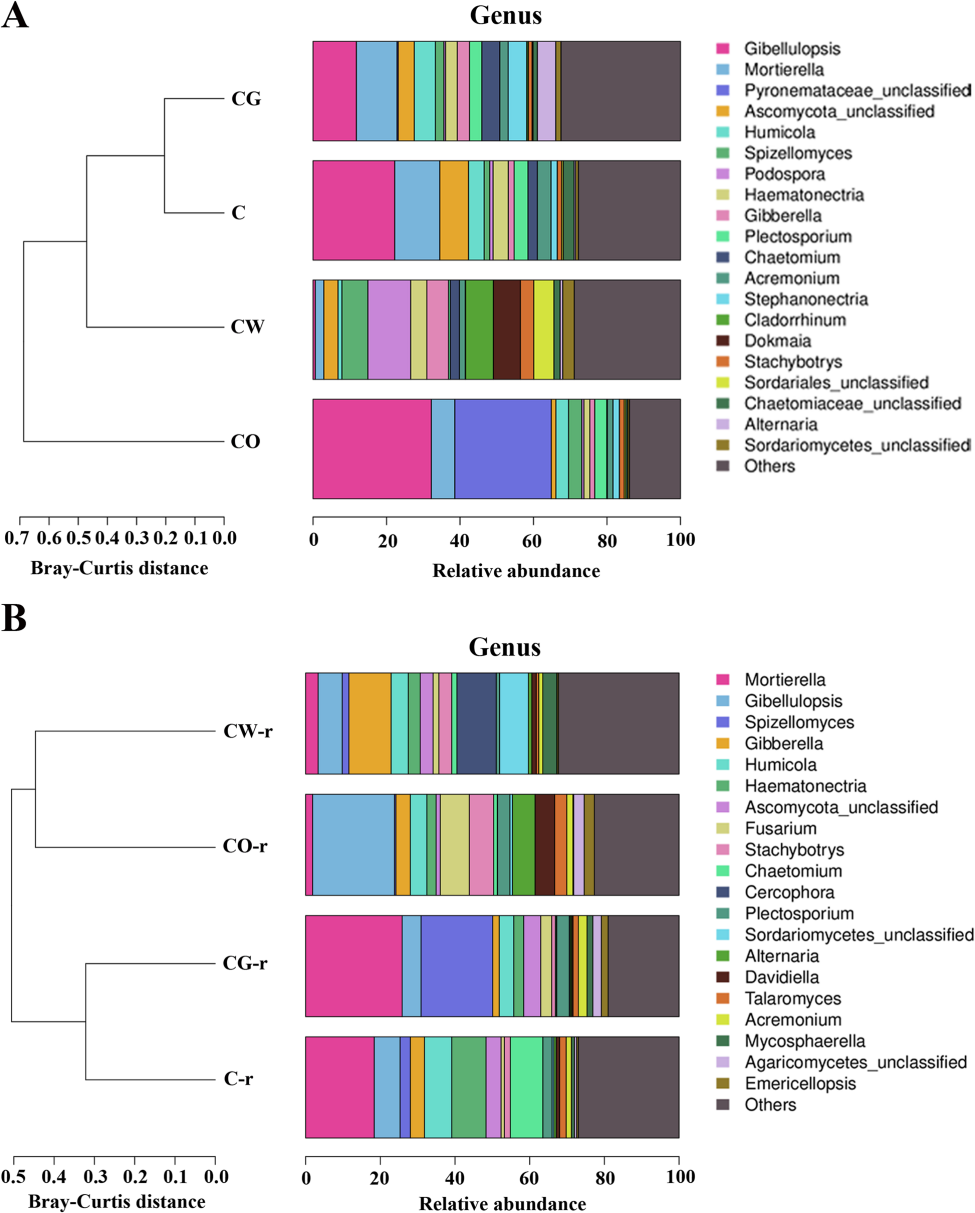
Classification of soil fungal communities in different planting patterns (at the genus level). A represented the fungal communities in the soil before planting, and B represented the fungal communities in the rhizosphere soil of plants infected by *V. dahliae*. C represented cotton monoculture, CW represented cotton-wheat intercropping, CG represented cotton-garlic intercropping, CO represented cotton-onion intercropping

For fungi, before crop planting, there were 5 genera with relative abundance of more than 4% in the cotton monoculture, namely *Gibellulopsis*, *Mortierella*, *Humicola*, *Haematonectria* and *Ascomycota*, accounting for 50.74% of the total flora; t﻿here were 7 genera in cotton-wheat intercropping, including *Podospora*, *Spizellomyces*, *Gibberella*, *Cladorrhinum*, *Haematonectria*, *Sordariales* and *Dokmaia*, accounting for 49.46% of the total flora; there were 3 genera in cotton-onion intercropping, namely *Gibellulopsis*, *Mortierella* and *Pyronemataceae*, accounting for 64.87% of the total flora; there were 7 genera in cotton-garlic intercropping, including *Gibellulopsis*, *Mortierella*, *Humicola*, *Alternaria*, *Stephanonectria*, *Chaetomium* and *Ascomycota*, accounting for 47.90% of the total flora. The dominant fungal community changes the most after cotton-wheat intercropping. In the cotton garlic-intercropping, the abundance of *Chaetomium* accounted for 4.90% of the total fungal population, and the abundance was the highest in these four plots, almost twice that of cotton monoculture and cotton-wheat intercropping, and 24.5 times that of cotton-onion intercropping (Fig. 7). The dominant microorganism (Top 5) in the four planting patterns was showed in Table 1.

**Table 1:** The dominant microorganism in the four planting patterns at the genus level. CK represents the soil samples before cotton planting, and Rhizosphere represent the rhizosphere soil samples of diseased plants. C represented cotton monoculture, CW represented cotton-wheat intercropping, CG represented cotton-garlic intercropping, CO represented cotton-onion intercropping

	Planting modes	Dominant microorganisms (Top 5)	
		Fungi	Bacteria
CK	C	*Gibellulopsis*、*Mortierella*、*Ascomycota*、*Humicola、Haematonectria*	*Gp6*、Bacteria_unclassified、*Gemmatimonas*、*Sphingomonas、Pontibacter*
	CW	*Podospora*、*Spizellomyces*、*Gibberella*、*Cladorrhinum、Dokmaia*	*Gp6*、Bacteria_unclassified、*Gemmatimonas*、*Sphingomonas、Pseudomonas*
	CO	*Gibellulopsis*、*Mortierella*、*Pyronemataceae*、*Humicola*、*Spizellomyces*	*Gp6*、Bacteria_unclassified、*Gemmatimonas、Gp4、Pseudomonas*
	CG	*Gibellulopsis*、*Mortierella*、*Humicola*、*Alternaria*、*Stephanonectria*	*Gp6*、Bacteria_unclassified、*Gemmatimonas*、*Sphingomonas、Pseudomonas*
Rhizosphere	C	*Mortierella、Gibellulopsis*、*Humicola、Haematonectria、Chaetomium*	*Gp6*、Bacteria_unclassified、*Gemmatimonas*、*Sphingomonas、Gp4*
	CW	*Gibellulopsis、Gibberella、Humicola、Cercophora、Sordariomycetes*	*Gp6*、Bacteria_unclassified、*Gemmatimonas*、*Sphingomonas、Gp4*
	CO	*Gibellulopsis、Fusarium、Stachybotrys、Alternaria、Davidiella*	*Gp6*、Bacteria_unclassified、*Gemmatimonas*、*Sphingomonas、Gp4*
	CG	*Mortierella、Gibellulopsis*、*Spizellomyces、Humicola、Ascomycota*	*Gp6*、Bacteria_unclassified、*Gemmatimonas*、*Sphingomonas、Gp4*

During the onset of Cotton Verticillium wilt, the soil rhizosphere fungal community has changed. There were 6 genera with relative abundance of more than 4% in the cotton monoculture, of which 5 genera were the same as before planting, and *Chaetomium* was added, accounting for 54.51% of the total flora. There were 5 genera in cotton-wheat interplants, namely, *Gibellulopsis*, *Humicola*, *Gibberella*, *Sordariomycetes* and *Cercophora*, accounting for 40.59% of the total flora. There were 6 genera in cotton-onion intercropping, namely *Gibellulopsis*, *Humicola*, *Fusarium*, *Stachybotrys*, *Alternaria* and *Davideiella*, accounting for 51.96% of the total flora. There were 4 genera in cotton-garlic intercropping, namely *Gibellulopsis*, *Mortierella*, *Spizellomyces* and *Ascomycota*, accounting for 54.67% of the total flora (Fig. 7).

**Fig. 8:**
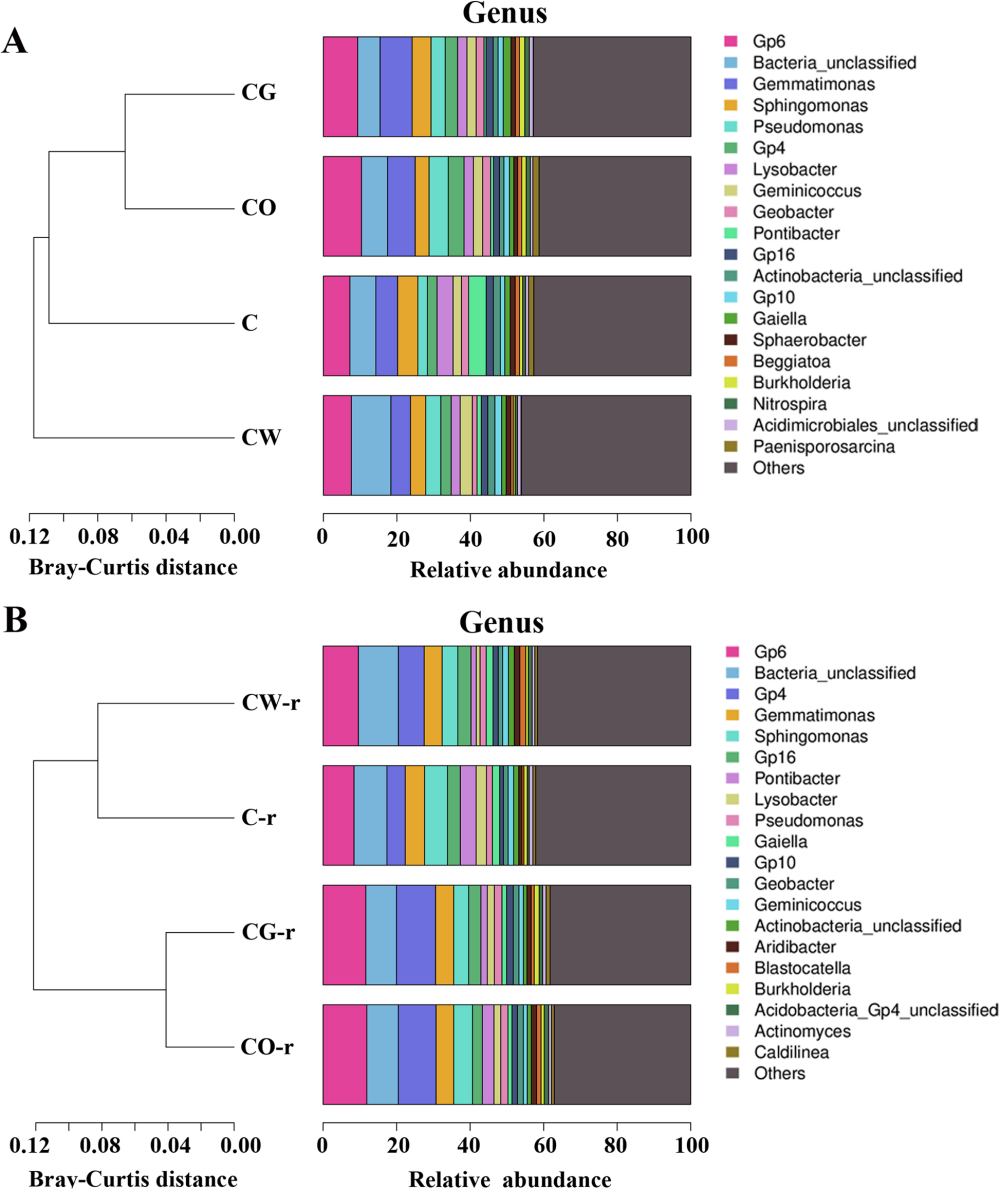
Classification of soil bacterial communities in different planting patterns (at the genus level). A represented the bacterial in the soil before planting, and B represented the bacterial in the rhizosphere soil of diseased plants during the occurrence period of Cotton Verticillium wilt after planting. C represented cotton monoculture, CW represented cotton-wheat intercropping, CG represented cotton-garlic intercropping, CO represented cotton-onion intercropping

For bacteria, before planting, the dominant bacteria in the four planting patterns were basically the same. The dominant bacteria in cotton-onion interplanting, cotton garlic-interplanting and cotton-wheat interplanting were *Gp6* and *Bacteria_ Unclassified*, *Gemmatimonas*, *Sphingomonas* and *Pseudomonas.* The dominant bacteria in cotton monoculture were *Gp6* and *Bacteria_ Unclassified*, *Gemmatimonas*, *Sphingomonas* and *Pontibacter*. Compared with intercropping, *Pseudomonas* was not the dominant bacteria in the cotton monoculture. The cotton-onion intercropping and the cotton monoculture contained *Paenisporosarcina*, while the other two patterns did not. The proportion of *Paenisporosarcina* in the cotton-onion intercropping was greater than that in the cotton monoculture. During the onset of Cotton Verticillium wilt, the first five dominant bacteria in these four patterns were the same, and only one genus of each pattern had changed compared with that before planting (Fig. 8, Table 1). The results showed that compared with the cotton monoculture, *Pseudomonas* was the dominant flora in the cotton-onion, cotton-garlic and cotton-wheat intercropping patterns.

### Differential microbial communities in the four different planting patterns

Before planting, the abundance of Cyanobacterium phylum in cotton monoculture plots was significantly higher than that in the other three plots, and the abundance of Actinobacteria phylum in cotton rhizosphere soil was significantly higher than that in cotton-onion intercropping plots (Data not showed).

**Fig. 9:**
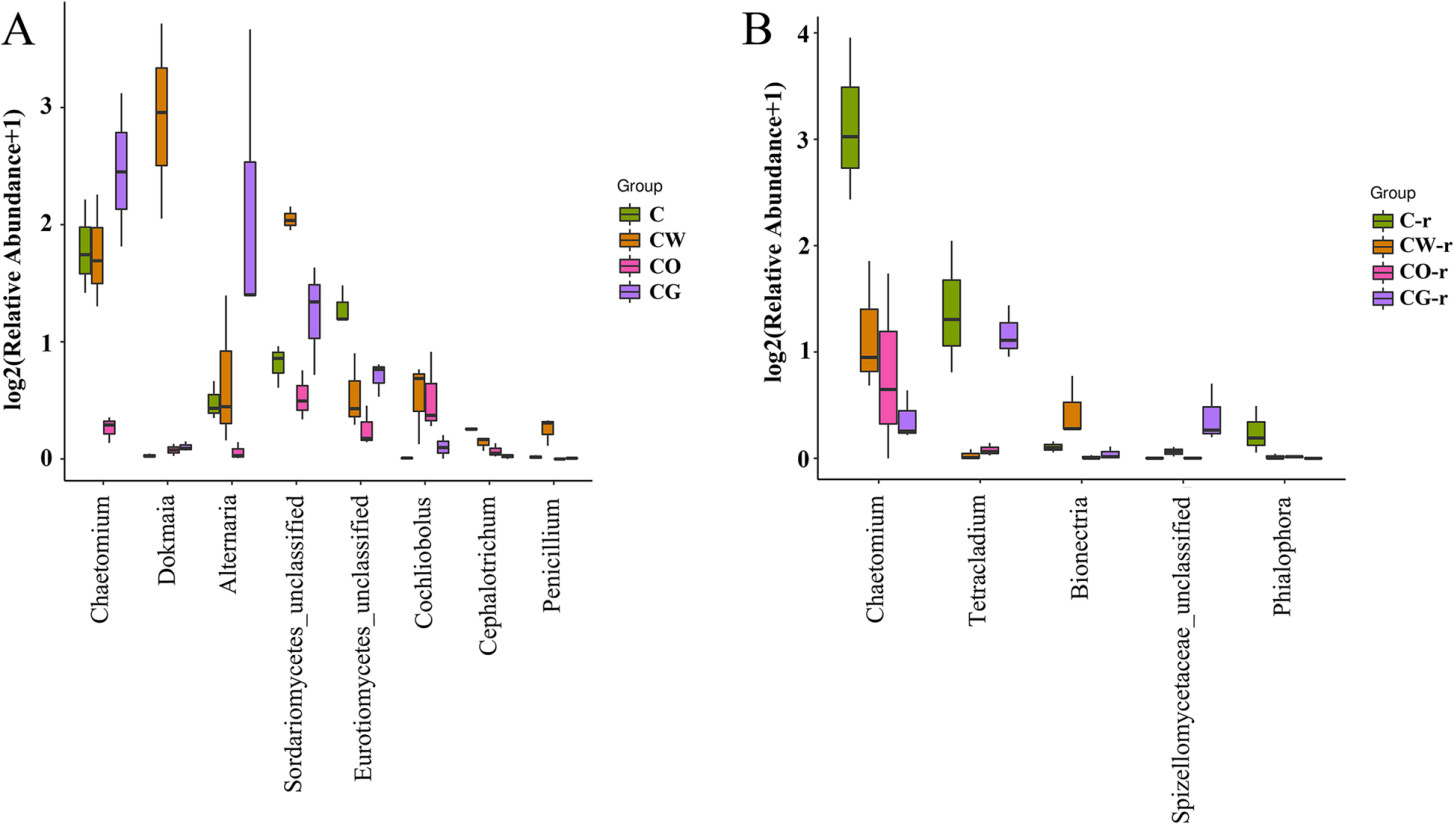
Differential abundance of soil fungal communities in different planting patterns (at genus level). A represented the different fungal communities in the soil before planting, B represented the different fungal communities in the rhizosphere soil of plants infected by *V. dahliae*. C represented cotton monoculture, CW represented cotton-wheat intercropping, CG represented cotton-garlic intercropping, CO represented cotton-onion intercropping

Before planting, the fungi with different abundance in the four planting patterns include *Chaetomium*, *Dokmaia*, *Alternaria*, *Sordariomycetes*, *Eurotiomycoetes*, *Cochliobolus*, *Cephalotrichum*, *Penicillium,* etc. (Table 2). In the cotton-garlic intercropping pattern, *Chaetomium* and *Alternaria* had the highest abundance. *Dokmaia*, *Sordariomycetes*, *Cochliobolus* and *Penicillium* had the highest abundance in the cotton-wheat intercropping, and *Sordariomycetes* had the lowest abundance in the cotton-onion intercropping (Fig. 9, Table 3). During the onset of Verticillium wilt, the differential flora also changed, including *Chaetomium*, *Tetracladium*, *Bionectria*, *Phyllophora* and *Spicellomyceteceae* (Fig. 9, Table 2). Among them, *Chaetomium*, *Tetracladium* and *Phyllophora* had the highest abundance in the rhizosphere of cotton monoculture, *Bionectria* had the highest abundance in cotton-wheat intercropping, and *Spicellomyceteceae* had the highest abundance in cotton-garlic intercropping (Fig. 9, Table 3).

**Table 2:** Fungi and bacteria with significant different abundances in the four planting patterns at the genus level (Top 15). CK represents the soil samples before cotton planting, and Rhizosphere represent the rhizosphere soil samples of diseased plants. The difference was analysis at *p* < 0.05

Fungi (genus)		Bacteria (genus)	
CK	Rhisphere	CK	Rhisphere
*Candida*	*Edenia*	*Psychroflexus*	*Marinobacterium*
*Hyponectriaceae*	*Claroideoglomus*	*Hoeflea*	*Sediminibacter*
*Olpidium*	*Occultifur*	*Parvularcula*	*Wenzhouxiangella*
*Acrostalagmus*	*Candida*	*Muricauda*	*Alistipes*
*Periconia*	*Meyerozyma*	*Sediminibacter*	*Chromatocurvus*
*Natantispora*	*Myrmecridium*	*Sphingomicrobium*	*Pelagibius*
*Volutella*	*Spizellomycetaceae*	*Ectothiorhodospira*	*Cupriavidus*
*Strigula*	*Magnaporthaceae*	*Elioraea*	*Candidatus_Solibacter*
*Alternaria*	*Tetracladium*	*Parapusillimonas*	*Gp5*
*Embellisia*	*Phialophora*	*Catalinimonas*	*Edaphobacter*
*Setosphaeria*	*Chaetomium*	*Thioalkalispira*	*Ochrobactrum*
*Meyerozyma*	*Bionectria*	*Sphingobacterium*	*Burkholderia*
*Magnaporthaceae*	*Myceliophthora*	*Mucilaginibacter*	*Aciditerrimonas*
*Stemphylium*	*Hypocrea*	*Gp26*	*Carboxydothermus*
*Cephalotrichum*	*Ceratobasidium*	*Cytophagales*_unclassified	*Cytophagales*_unclassified

**Table 3:** The abundances of beneficial microorganism in the four planting patterns at the genus level. CK represents the soil samples before cotton planting, and Rhizosphere represent the rhizosphere soil samples of diseased plants. Bold font represents the highest value

	Genus	C	CW	CO	CG
CK	*Burkholderia*	0.84	0.50	1.22	**1.46**
	*Dokmaia*	0.02	**7.36**	0.06	0.08
	*Penicillium*	0.01	**0.19**	0.00	0.01
	*Chaetomium*	2.55	2.49	0.20	**4.90**
Rhizosphere	*Chaetomium*	**8.68**	1.39	0.97	0.31
	*Bionectria*	0.08	**0.38**	0.01	0.03
	*Burkholderia*	0.92	0.71	0.85	**1.37**

Before planting, the bacteria with different abundance mainly included *Pontibacter*, *Burkholderia*, *Nitrospira*, *Skermanellah*, *Mesorhizobium*, etc. (Table 2). The abandance of *Burkholderia* in cotton-onion and cotton-garlic intercropping was significantly higher than that in cotton monoculture (Table 3). At the onset stage of Cotton Verticillium wilt, different bacterial communities included *Gp4*, *Geobacter*, *Blastocatella*, *Burkholderia*, *Gp3*, etc. (Table 2). In the cotton-garlic intercropping, the abandance of *Burkholderia* was significantly higher than that in the cotton monoculture (Table 3).

## Discussion

Verticillium wilt of cotton is a serious soil-borne disease that seriously affects the yield and quality of cotton. Due to increased environmental awareness and the need for sustainable cotton production, the use of intercropping to control cotton diseases is receiving increasing attention [28]. Intercropping garlic with cotton is currently one of the main cropping systems in China [29]. Cotton-cereals intercropping can reduce the number of pathogens and reduce the incidence and severity of cotton diseases [28]. Wheat-cotton, cabbage-cotton, watermelon-cotton and melon-cotton intercropping can help reduce boll rot [30, 31]; cotton-peanut intercropping can effectively control *Meloidogyne incognita* [32, 33]. Root secretions of intercropping plants usually inhibit the proliferation of pathogens. Garlic root secretions can inhibit the proliferation of cotton pathogens [34], and cotton Fusarium wilt and Verticillium wilt were significantly reduced after intercropping with garlic. Carrot root secretions have a strong inhibitory effect on cotton damping off [35]. In this study, cotton-garlic and cotton-onion intercropping significantly reduced the disease index of Verticillium wilt. Disease index decreased in cotton-wheat intercropping but was not significantly different compared to cotton monocropping. The seedling disease index was also relatively lower after intercropping, but not significantly different compared to cotton monoculture.

Intercropping can alter the composition of microbial communities. Maize and peanut intercropping increased the number and diversity of beneficial microorganisms in the soil and reduced pathogenic and toxic microorganisms [36, 37]. It was found that intercropped maize and peanut bacterial community shannon and Chao 1 indices decreased, community diversity and richness decreased, and intercropped peanut fungal community diversity and richness increased [38]; wheat-cucumber and mustard-cucumber intercropping systems increased soil microbial OTU abundance, fungal community diversity and bacterial community diversity [19]. In this study, intercropping also altered the diversity of the microbial communities. The fungal shannon index were significantly lower in the pre-planting cotton-onion intercropping plots than in the other three plots. The three indices Chao 1, shannon and Sobs of fungal and bacterial communities were highest in cotton-garlic intercropping plots and higher than in cotton monoculture plot; Chao 1, shannon and Sobs fungal indices were lowest in rhizosphere soil of cotton-onion intercropping plot at the onset of cotton Verticillium wilt. In this study, the index of Cotton Verticillium wilt disease in cotton-garlic and cotton-onion intercropping was significantly decreased, 45.46% in cotton-garlic intercropping pattern and 37.01% in cotton-onion intercropping pattern, and the index of Cotton Verticillium wilt disease in cotton-wheat intercropping pattern was also reduced, but the difference was not significant. Increased fungal and bacterial diversity in cotton-garlic plot and reduced fungal diversity in cotton-onion plot may be associated with reduced Cotton Verticillium wilt disease index in cotton.

Soil microorganisms can affect the occurrence of diseases, and beneficial soil microorganisms can help resist pathogens [39, 40]. Intercropping promotes the accumulation of beneficial bacteria and thus resistance to pathogenic infections. The relative abundance of beneficial microorganisms such as *Bacillales*, *Pseudomonas*, *Rhizobiales* and *Trichoderma* in rhizosphere and endophyte of cotton varieties resistant to Verticillium wilt was higher than that of susceptible varieties [22]; the soil bacterial community structure differed more between tobacco-peanut intercropping and peanut continuous cropping, with an increased proportion of beneficial bacteria *Bacillus* and *Lactococcus* [41]. There were some beneficial bacteria, such as *Chaetomium* sp., in the lily rhizosphere soil in the interplanting pattern that had not existed in the single-cropping pattern [42]. *Chaetomium globosum* can produce a variety of secondary metabolites to control plant diseases [43–45], including Cotton Verticillium wilt [46]. *C. globosum* CEF-082 also increased the resistance of cotton to Verticillium wilt [47]. In this study, before planting, *Chaetomium* was the highest in the cotton-garlic intercropping pattern, *Penicillium* had the highest abundance in the cotton-wheat intercropping, and the abundance of *Burkholderia* in the cotton-garlic and cotton-onion intercropping patterns were significantly higher than that in the cotton monoculture pattern; the abundance of *Burkholderia* in the cotton-garlic intercropping pattern was still significantly higher than that in the monoculture pattern during the onset of Cotton Verticillium wilt. Before planting, *Pseudomonas* was the dominant microorganism in the cotton-onion intercropping, cotton-wheat intercropping and cotton-garlic intercropping patterns, while *Pseudomonas* was not the dominant bacteria in the cotton monoculture. Four beneficial microorganisms, *Chaetomium*, *Penicillium, Pseudomonas* and *Burkholderia*, were presumed to play an important role in suppressing the development of Cotton Verticillium wilt. *Chaetomium, Penicillium* and *Pseudomonas* might inhibit the occurrence of diseases by producing secondary metabolites that inhibited the growth of *V. dahliae.* For example, *Chaetomium* could produce chaetoviridin A to inhibit the growth of *V. dahliae* Vd080 [46]; *Penicillium* produced penicillic acid, which could effectively suppress the development of fire blight disease [48]; Nunamycin, nunapeptin, brasmycin and braspeptin produced by *Pseudomonas* had antifungal activity [49]. *Chaetomium* might also enhance the resistance of cotton to Verticillium wilt. *Burkholderia* could improve the resistance of soybean to aluminum stress [50], and the control effectiveness of *Burkholderia* on Cotton Verticillium wilt needs further study.

## Conclusion

This study confirmed that intercropping affected the composition of the soil microbial community and suppressed the occurrence of the soil-borne disease Verticillium wilt. Before planting, soil fungal diversity was significantly lower in the cotton and onion intercropping plot than in the other three plots. The highest abundance of *Chaetomium* and *Burkholderia* was found in the in cotton-garlic intercropping plot and the highest abundance of *Penicillium* in the cotton-wheat intercropping plot. The disease index of all three intercropping plots was lower than that of cotton monoculture plots, while the disease index of the cotton-garlic and cotton-onion intercropping was significantly lower than that of the cotton monoculture plots. Therefore, these factors should be related to the occurrence of Verticillium wilt, and the disease is a combination of these factors.

## Data Availability

Data of ITS and 16S amplicon sequencing of the samples were submitted to NCBI under the Bioproject ID PRJNA891938 (https://www.ncbi.nlm.nih.gov/bioproject/?term=PRJNA891938) and PRJNA892167 (https://www.ncbi.nlm.nih.gov/bioproject/?term=PRJNA892167), respectively.

## Abbreviations

PAL: Phenylalanine ammonia lyase
PPO: Polyphenol oxidase
CW: Cotton-wheat interplanting
CG: Cotton-garlic intercropping
CO: Cotton-onion interplanting
C: Cotton monoculture
OTUs: Operational taxonomic units
Sobs: Observed species
PCoA: Principal coordinate analysis
ANOVA: One-way analysis of variance
